# Neurophysiological Correlates of Asymmetries in Vowel Perception: An English-French Cross-Linguistic Event-Related Potential Study

**DOI:** 10.3389/fnhum.2021.607148

**Published:** 2021-06-03

**Authors:** Linda Polka, Monika Molnar, T. Christina Zhao, Matthew Masapollo

**Affiliations:** ^1^School of Communication Sciences and Disorders, McGill University, Montreal, QC, Canada; ^2^Center for Research on Brain, Language, and Music, McGill University, Montreal, QC, Canada; ^3^Department of Speech-Language Pathology, University of Toronto, Toronto, ON, Canada; ^4^Institute for Learning and Brain Sciences, University of Washington, Seattle, WA, United States; ^5^Department of Speech and Hearing Sciences, University of Washington, Seattle, WA, United States; ^6^Department of Speech, Language, and Hearing Sciences, University of Florida, Gainesville, FL, United States

**Keywords:** vowel perception, mismatch negativity, prototypes, natural referent vowel framework, native language magnet model, brain rhythms

## Abstract

Behavioral studies examining vowel perception in infancy indicate that, for many vowel contrasts, the ease of discrimination changes depending on the order of stimulus presentation, regardless of the language from which the contrast is drawn and the ambient language that infants have experienced. By adulthood, linguistic experience has altered vowel perception; analogous asymmetries are observed for non−native contrasts but are mitigated for native contrasts. Although these directional effects are well documented behaviorally, the brain mechanisms underlying them are poorly understood. In the present study we begin to address this gap. We first review recent behavioral work which shows that vowel perception asymmetries derive from phonetic encoding strategies, rather than general auditory processes. Two existing theoretical models–the Natural Referent Vowel framework and the Native Language Magnet model–are invoked as a means of interpreting these findings. Then we present the results of a neurophysiological study which builds on this prior work. Using event-related brain potentials, we first measured and assessed the mismatch negativity response (MMN, a passive neurophysiological index of auditory change detection) in English and French native-speaking adults to synthetic vowels that either spanned two different phonetic categories (/y/vs./u/) or fell within the same category (/u/). Stimulus presentation was organized such that each vowel was presented as standard and as deviant in different blocks. The vowels were presented with a long (1,600-ms) inter-stimulus interval to restrict access to short-term memory traces and tap into a “phonetic mode” of processing. MMN analyses revealed weak asymmetry effects regardless of the (i) vowel contrast, (ii) language group, and (iii) MMN time window. Then, we conducted time-frequency analyses of the standard epochs for each vowel. In contrast to the MMN analysis, time-frequency analysis revealed significant differences in brain oscillations in the theta band (4–8 Hz), which have been linked to attention and processing efficiency. Collectively, these findings suggest that early-latency (pre-attentive) mismatch responses may not be a strong neurophysiological correlate of asymmetric behavioral vowel discrimination. Rather, asymmetries may reflect differences in neural processing efficiency for vowels with certain inherent acoustic-phonetic properties, as revealed by theta oscillatory activity.

## Introduction

A central goal of research in the field of speech perception is to explicate how listeners map the input acoustic signal onto the phonetic categories of language (for reviews, Cleary and Pisoni, 2001; Fowler, 2003; Diehl et al., 2004; Samuel, 2011). Within this overarching agenda, developmentalists have addressed how this mapping between acoustic and phonetic structures dynamically changes via early language experience in the first year of life (Werker and Curtin, 2005; Kuhl et al., 2008; Best et al., 2016). This emphasis on describing infant attunement to native speech derived in large part from experimental investigations by Kuhl and colleagues (Grieser and Kuhl, 1989; Kuhl, 1991; Kuhl et al., 1992; Iverson and Kuhl, 1995, 2000; Iverson et al., 2003). Their studies with human infants, human adults, and rhesus macaques revealed that early language experience profoundly alters speech perception by reducing discrimination sensitivity close to phonetic category prototypes and boosting sensitivity at the boundaries between categories (Lotto et al., 1998; Guenther et al., 1999, 2004; Feldman et al., 2009).

In more recent years, however, it has become increasingly clear that from infancy onward, speech processing involves generic as well as language-specific perceptual biases. It is now known that infants from across diverse linguistic communities initially display generic, “language-universal” biases or preferences in their perception of phonetic segments (Polka and Bohn, 2003, 2011; Nam and Polka, 2016). Moreover, these generic or “all-purpose” speech biases, which are distinct from “language-specific” prototype categorization processes, have been identified in adults (Masapollo et al., 2017a; Liu et al., 2021). These generic speech biases are evident in studies showing that young infants exhibit robust listening preferences for some speech sounds over others (Polka and Bohn, 2011; Nam and Polka, 2016), and that some phonetic contrasts are poorly distinguished early on (Polka et al., 2001; Best and McRoberts, 2003; Larraza et al., 2020) or show directional asymmetries in discrimination (Polka and Bohn, 2003, 2011; Kuhl et al., 2006; Pons et al., 2012; Nam and Polka, 2016).

The present research aims to improve our understanding of the neural mechanisms and processes underlying vowel perception biases observed in adults. It has been known for years that, early in development, infant perception is biased toward articulatorily and acoustically extreme vowels. These findings have been reviewed and discussed extensively by Polka and Bohn (2003, 2011), and have also been reinforced in recent meta-analyses (Tsuji and Cristia, 2017; Polka et al., 2019). Evidence supporting this view initially emerged from research revealing that infants show robust directional asymmetries in vowel discrimination tasks. More specifically, infants perform better at discriminating a change from a relatively less peripheral to a relatively more peripheral vowel within *F*_1_–*F*_2_ acoustic space, regardless of the language from which the contrast is drawn. As an example, Bohn and Polka (2001) used the head-turn conditioning procedure to test German-learning infants’ discrimination of the German/i/-/e/vowel contrast (Werker et al., 1998). In this task, infants hear a repeating background stimulus and are assessed on their ability to distinguish a change from the background to a target stimulus. In the Bohn and Polka study, they counterbalanced presentation of each vowel; half of infant subjects were tested with one direction of change (from /i/ to /e/) and half were tested with a change in the reverse direction (from /e/ to /i/). The results revealed that infants performed better at discriminating the change from /i/ to /e/, compared to the reverse. Similar directional effects have been found with infants tested using numerous behavioral tasks and a wide range of vowel contrasts (Polka and Bohn, 2003, 2011). By adulthood, linguistic experience has altered vowel perception; similar asymmetries are observed for other non−native contrasts but not for native contrasts which are typically perceived with near-perfect accuracy (Polka and Bohn, 2011; Dufour et al., 2013; Tyler et al., 2014).

Over the last decade, Polka and colleagues have formulated and experimentally tested a theoretical framework, termed the Natural Referent Vowel (NRV) framework, for explicating the processes underlying directional asymmetries (Polka and Bohn, 2011; Masapollo et al., 2017a, 2018a; Polka et al., 2019). The NRV framework incorporates ideas across several existing phonetic theories, namely Steven’s Quantal Theory (Stevens, 1989), and Schwartz’s Dispersion-Focalization Theory (Schwartz and Escudier, 1989; Schwartz et al., 1997, 2005). Quantal Theory posits that vocalic articulations affiliated with the extremes of vowel space result in acoustic signals with obvious spectral prominences created by the convergence of adjacent formant frequencies. For example, when producing /i/ (the highest front vowel) *F*_2_, *F*_3_, and *F*_4_ converge, when producing /y/ (the highest front rounded vowel) *F*_2_ and *F*_3_ converge, when producing /a/ (the lowest back vowel) and /u/ (the highest back vowel) *F*_1_ and *F*_2_ converge. These convergence points have also been referred to as “focal points” (Boë and Abry, 1986). According to the Dispersion-Focalization Theory, the strong tendency for vowel systems to select members found at the extremes of articulatory/acoustic vowel space is driven by two factors. First, dispersion ensures that vowels are acoustically distant from one another within vowel space, which enhances perceptual differentiation. Second, focalization ensures that vowels have salient and stable phonetic structures making them strong anchors for perception and production. Focal vowels will be easier for listeners to detect, encode, and retain in phonological working memory.

Concurring with these fundamental principles, the NRV framework (Polka and Bohn, 2011) offers additional insights into the aforementioned developmental findings by proposing that asymmetries in infant and adult vowel discrimination reflect a default, generic perceptual bias favoring focal vowels. In this account, the focalization of acoustic energy boosts perceptual salience, which in turn, biases perception and gives rise to the directional asymmetries observed in phonetic discrimination tasks (Schwartz and Escudier, 1989; Masapollo et al., 2017a). In advancing this viewpoint, Polka and Bohn do not mean to imply that perceptual asymmetries are attributable to low-level auditory or psychoacoustic processes. As highlighted in Masapollo et al. (2017b, 2018a), *NRV assumes that the effects of formant convergence on vowel perception reflect a phonetic bias that emerges when listeners are perceiving speech*, *rather than a low-level sensitivity to raw acoustic energy*. Compatible with this view, perception experiments have demonstrated that asymmetries predicted by differences in formant proximity are observed whether vowels are heard or perceived visually in a lip-reading task (Masapollo et al., 2017b, 2018a; Masapollo and Guenther, 2019), confirming that the “focal vowel” bias derives from phonetic processing rather than low-level psychoacoustic processes (Masapollo et al., 2019).

Polka and Bohn (2011) have further argued that the focal vowel bias plays an important role in the acquisition and processing of vowels across the lifespan. Asymmetries that point to a focalization bias are observed in infants in the first few months of life for both native and non-native vowel contrasts alike. Across the first year, as infants accrue specific linguistic experience, they begin tuning to native vowel contrasts. This will increase or diminish the initial focalization bias depending on the vowel inventory of their native language. This generic bias is thought to provide a scaffold to support the acquisition of a more detailed vowel system. Thus, according to NRV, both generic/focalization biases and language-specific biases influence vowel perception in mature, adult language users.

An alternative, but not mutually exclusive, account of asymmetries derives from Kuhl’s Native Language Magnet (NLM) model (Kuhl, 1991; Iverson and Kuhl, 1995; Kuhl et al., 2008). This model, which combines principles from categorization and prototype theory (Rosch, 1975, 1977; Samuel, 1982) with statistical learning theory (Aslin and Newport, 2012), posits that directional asymmetries reflect biases favoring native language phonetic category prototypes (i.e., adult-defined “best” instances of a category). NLM assumes that phonetic categories emerge early in development as infants track distributional patterns in speech input during social interactions. Like other cognitive/perceptual categories, phonetic categories have an internal structure organized around a central, prototypic member (Kuhl, 1991). Furthermore, Kuhl claims that these prototypes have a “magnet-like” effect, which shrinks the immediate perceptual space making it more difficult to discriminate variants surrounding a prototype compared to variants surrounding a non-prototype of the same category (Kuhl, 1991; Kuhl et al., 1992; Iverson and Kuhl, 1995; cf. Miller and Eimas, 1996; Guenther et al., 1999, 2004; Feldman et al., 2009). Although NLM applies to both consonants and vowels, most of the research supporting the idea that there is a “warping” of within-category perceptual space that is tied to variation in category goodness has focused on vowels (Kuhl, 1991; Kuhl et al., 1992; Iverson and Kuhl, 1995; Lotto et al., 1998). Moreover, NLM posits that speech perception relies on general auditory mechanisms applied to acoustic rather than specifically phonetic information. Nevertheless, in the NLM model, directional asymmetries are viewed as an experience-dependent bias favoring native prototype; asymmetries arise because listener sensitivity is reduced when discriminating a change from a more-prototypic to a less-prototypic vowel compared to the reverse. In line with this view, Kuhl (1991) reported a directional asymmetry in which English-learning infants performed better at discriminating a change from a non-prototypic /i/ to a prototypic /i/, compared to a change from prototypic /i/ to non-prototypic /i/. Notably, in this case the prototypic /i/ was more focal (between *F*_2_ and *F*_3_) compared to the non-prototypic /i/. Thus, the observed asymmetry could be due to prototypicality and/or focalization effects.

Several English-French cross-linguistic studies assessed the competing NRV and NLM accounts of asymmetries in vowel perception (Masapollo et al., 2017a,b; Liu et al., 2021). The vowel /u/, as in *boo*, was chosen for use in these studies for several reasons. First prior research established that Canadian French speakers consistently produce more extreme /u/ gestures (resulting in lower and spectrally closer *F*_1_ and *F*_2_ values) than Canadian English speakers. Accordingly, in the standard vowel space, the mean location of French /u/ is more peripheral than that of English /u/ (Escudero and Polka, 2003; MacLeod et al., 2009; Noiray et al., 2011). This means that French /u/ has a more focal acoustic-phonetic form (with closer convergence of *F*_1_ and *F*_2_) compared to English /u/. These differences in focalization and language-specific phonetic categorization between English and French speakers provided an ideal opportunity to assess how these factors influence adult vowel perception.

In an initial study, Masapollo et al. (2017a) synthesized a set of vowels that were consistently identified as /u/ by native speakers of English and of French but that nevertheless varied in their stimulus goodness ratings, such that the best French /u/ exemplars were more focal (between *F*_1_ and *F*_2_) compared to the best English /u/ exemplars. In an AX (same/different) discrimination task, both English and French listeners were found to perform better at discriminating changes from the less to the more focal /u/ compared to the reverse, regardless of variation in prototypicality. Similar results were obtained using natural productions of English /u/ and French /u/ in tests with adults (Masapollo et al., 2017b) and infants (Polka et al., 2018). These findings established the focal vowel bias in adults, demonstrated that this perceptual bias favors vowels with greater formant convergence and established that this bias operates independently of biases related to language-specific prototype categorization.

In a subsequent study, Liu et al. (2021) presented Canadian English listeners with a finer grained series of vowels varying from the less-focal/English prototypic /u/ to the more-focal/French prototypic /u/ identified in the prior Masapollo et al. (2017a) study. In an AX discrimination task, the stimulus pairings included one-step, two-step, and three-step intervals along the series. The results revealed that focalization and prototype effects were both present but were differentially influenced by the size of the acoustic intervals along the stimulus series. More specifically, asymmetries favoring the English /u/ prototype emerged when subjects were discriminating small stimulus differences (1-step) close to the prototype stimulus. When stimulus differences were larger (2- or 3-steps) discrimination asymmetries favored more focal exemplars of /u/ (Masapollo et al., 2015). Collectively, these findings demonstrate, at the behavioral level, that directional asymmetries in adult vowel perception reveal a generic “focal vowel” bias that shapes the global structure of the vowel space (explained by NRV) as well as a more subtle experience-dependent bias that alters perception of the local internal structure of native vowel categories (as described by NLM).

Although the existing behavioral data indicate that directional asymmetries may be well predicted from a combination of salient spectral information and category “goodness” ratings, the neural underpinnings of these effects remain to be determined. Here, we present data from neurophysiological experiments with adults from different language backgrounds to begin to uncover these “brain-to-perception” relations and generate new hypotheses within the NRV and NLM theoretical frameworks. We wish to provide data that help to characterize what aspects of neural processing corroborate extant behavioral findings. Toward this end, we investigated whether we can observe asymmetries in the neurophysiological correlates of adult vowel perception, focusing on two neural measurements at the cortical level: (1) the mismatch negativity (MMN) that indexes neural sensitivity to vowel change; and (2) brain oscillatory activity in the theta (4–8 Hz) frequency band that indexes processing efficiency. While focalization biases have always been tested and demonstrated behaviorally by directional effects in discrimination tasks, examining the neural responses to vowels may provide us with a new window to understand vowel processing and the representation of “central” versus “peripheral” vowels in a more direct manner.

We recorded auditory event-related potentials (ERPs) and first computed the MMN response to within-category and cross-category vowel contrasts in native English- and French-speaking listeners. The vowel stimuli were previously used in an ERP study (Molnar et al., 2013) that compared vowel processing in bilingual and monolingual adults. The experimental design of this study also permitted an exploration of perceptual asymmetries, which is our present goal. Four stimuli were chosen from an acoustic vowel continuum (described below) ranging perceptually from /i/ to /y/ to /u/; the selected tokens include variants of /u/ and /y/ that form cross-category stimulus pairs (in French) and within-category stimulus pairs (in both languages). The psychophysical distances between the cross-category and within-category stimulus pairs were equated.

Prior studies examining the MMN in auditory oddball paradigms (Näätänen et al., 2007) have typically employed relatively short inter-stimulus-intervals (ISIs) (approximately 500 ms) in order to “build up” or “strengthen” the short-term memory “trace” for the repeated standard stimulus that develops online during the course of the experiment. The MMN is generally thought to reflect activity differences in neurons in or near the auditory cortex that detect a discrepancy (or mismatch) between the deviant percept and short-term trace of the standard (Näätänen et al., 2007). In tasks using relatively short ISIs, there will be less time for the short-term memory trace to decay between successive stimuli, and thus brain responses will reflect the basic resolution of the auditory system. Conversely, when the ISI is longer, the length of time that each stimulus is buffered in memory increases, short-term traces will decay, and brain responses will reflect stimulus encoding processing and long-term representations of phonological units (for discussion, see Strange, 2011). In the current research, we used a long ISI to better elicit a “phonetic mode” of processing and to restrict access to short-term memory traces. As previously discussed, NRV posits that focalization biases reflect phonetic processes rather than auditory processes in speech perception (Polka and Bohn, 2011; Masapollo et al., 2017a,b, 2018a,b). In keeping with this view, several previous behavioral studies have shown that in AX discrimination tasks, vowel order effects emerge or increase as the ISI increases, whereas overall perceptual performance improves and asymmetries decrease when the ISI decreases (Polka and Bohn, 2011; Masapollo et al., 2018b; Polka et al., 2019). For example, when testing adult discrimination of a non-native contrast, Polka and Bohn (2011) observed a directional asymmetry when they used a 1,500 ms ISI, but not when they used a 500 ms ISI.

On the basis of the aforementioned behavioral findings (Masapollo et al., 2017a; Liu et al., 2021), we generated several hypotheses concerning MMN responses measured using a long ISI. First, for relatively large (cross-category /u/ vs /y/) phonetic differences, we predicted (à la NRV) that the MMN will exhibit greater amplitude (and/or a shorter latency) in response to changes from less-focal to more-focal vowels compared to the reverse, but that these asymmetries will be weaker in French listeners because the /u/ - /y/ contrast is native in French but non-native in English. Second, for relatively small (within-category) phonetic differences, we hypothesized (à la NLM) that the MMN would exhibit greater amplitude (and/or a shorter latency) in response to changes from less-prototypic to more-prototypic vowels compared to the reverse. This hypothesis would be supported if the MMN showed opposite asymmetries across the two language groups. More specifically, English listeners would be expected to show a larger (and/or earlier) MMN when the English-prototypic /u/ occurs as a deviant among French-prototypic /u/ standards compared to the reverse, whereas French listeners would be expected to show a larger (and/or earlier) MMN when the French-prototypic /u/ occurs as a deviant among the English-prototypic /u/ standards compared to the reverse. Similarly, for the French group only, the MMN should be larger (and/or earlier) when the French-prototypic /y/ occurs as a deviant among the French-non-prototypic /y/ standards compared to the reverse. Yet another possibility is that directional asymmetries observed at the behavioral level may be reflected by ERP components with latency differences relative to the MMN. Because the MMN is thought to reflect “pre-attentive” processes, it may be too early of a cortical response to reflect asymmetries, which do not appear to derive from early stages of acoustic processing (Polka and Bohn, 2011; Masapollo et al., 2018b; Polka et al., 2019). Recently it has also been demonstrated that MMNs recorded in an oddball paradigm with a longer ISI (e.g., 600 vs. 2,600 ms) reflect sensitivity to language-specific phonological information rather than the acoustic information in speech sounds (Yu et al., 2017, 2018). An additional goal of the current study was to go beyond examination of the classic MMN response and track cortical oscillations. While the MMN response may seem like a more direct comparison with the existing behavioral discrimination findings, comparing the neural responses to the standard trials across the four vowels may provide us with a more direct look at how vowels with different acoustic characteristics are processed in the brain. We identified the theta band neural oscillation (4–8 Hz) to be a good measure to characterize vowel processing, as it has been argued to provide a measure of “neural efficiency” during speech processing (Zhang et al., 2011; Bosseler et al., 2013). We hypothesized that, if formant convergence influences attention or cognitive effort (à la NRV), then theta rhythms should show reduced power in response to more-focal compared to less-focal vowels. If, on the other hand, stimulus prototypicality influences cognitive effort (à la NLM), then theta rhythms should show reduced power in response to more prototypic native vowel exemplars compared to less prototypic ones.

## Materials and Methods

### Participants

Thirty normal-hearing right-handed adults participated in the current experiment: 15 were native Canadian English speakers (average age = 25, seven females) and 15 were native Canadian French speakers (average age = 26 years, seven females). All were healthy young adults with no history of a speech, language, or other neurological impairment. Informed consent was obtained according to the McGill University human research committee. Four additional participants were tested but excluded from the analysis due to technical problems with the data acquisition (2) and poor data quality caused by artifacts (2). The EEG/ERP data for these participants was collected in a previous study (Molnar et al., 2013).

Participants’ language background was assessed using two measures: (1) The *Language Experience and Proficiency Questionnaire* (LEAP-Q) which was developed specifically to evaluate bilingual and multilingual individuals’ linguistic experience (Marian et al., 2007); and (2) a speech sample evaluated by monolingual speakers of Canadian English (*n* = 3) and Canadian French (*n* = 3) using a scale from 1 (“no ability in the given language”) to 5 (“native-like ability”).

Participants had to meet the following criteria to be included in the study: (1) no prior linguistic or phonetics training; (2) raised in a monolingual home and educated in a monolingual school in their respective language; (3) no experience learning a second language before 10 years of age; (4) no experience conversing in a second language on a regular basis, having rated their speaking and listening abilities in a second language with a maximum of 4 out of 10 on the LEAP-Q; and (5) their speech samples were rated 5 (native-like) on average.

### Stimuli

In our previous behavioral studies (Molnar et al., 2010; Masapollo et al., 2017a), we synthesized a broad array of 128 vowels that covered the entire upper region of vowel space and ranged in *F*_1_ (from 275 to 330 Hz) and *F*_2_ (from 476 to 2,303 Hz) in equal psychophysical steps on the bark scale (Zwicker and Terhardt, 1980). All stimuli were synthesized using the Variable Linear Articulatory Model (Maeda, 1979, 1990; Boë, 1999; Ménard et al., 2004, 2009). The variants were created by manipulating the values of *F*_1_ and *F*_2_; the values of *F*_0_, *F*_3_, *F*_4_, and *F*_5_ remained constant for all vowels at 120, 2,522, 3,410, and 4,159 Hz, respectively. Each stimulus was 400 ms in duration and had the same intonation and intensity contours. In pilot studies, these stimuli were presented to native, monolingual Canadian English (*n* = 5) and Canadian French (*n* = 5) listeners, who were asked to give their phonetic identification and goodness ratings on a 5-point-scale (1 = very poor, 5 = very good). We found, as expected, that vowel judgments systematically varied as a function of *F*_2_: For English listeners, the vowels varied perceptually from /u/(“oo”) to /i/(“ee”) as *F*_2_ values increased, whereas for French listeners, the vowels varied perceptually from /u/(“oo”) to /y/ (as in the French word “but”) to /i/ (ee) as the *F*_2_ values increased (Note that in Canadian English, /y/ does not occur (Escudero and Polka, 2003; MacLeod et al., 2009).

**FIGURE 1:**
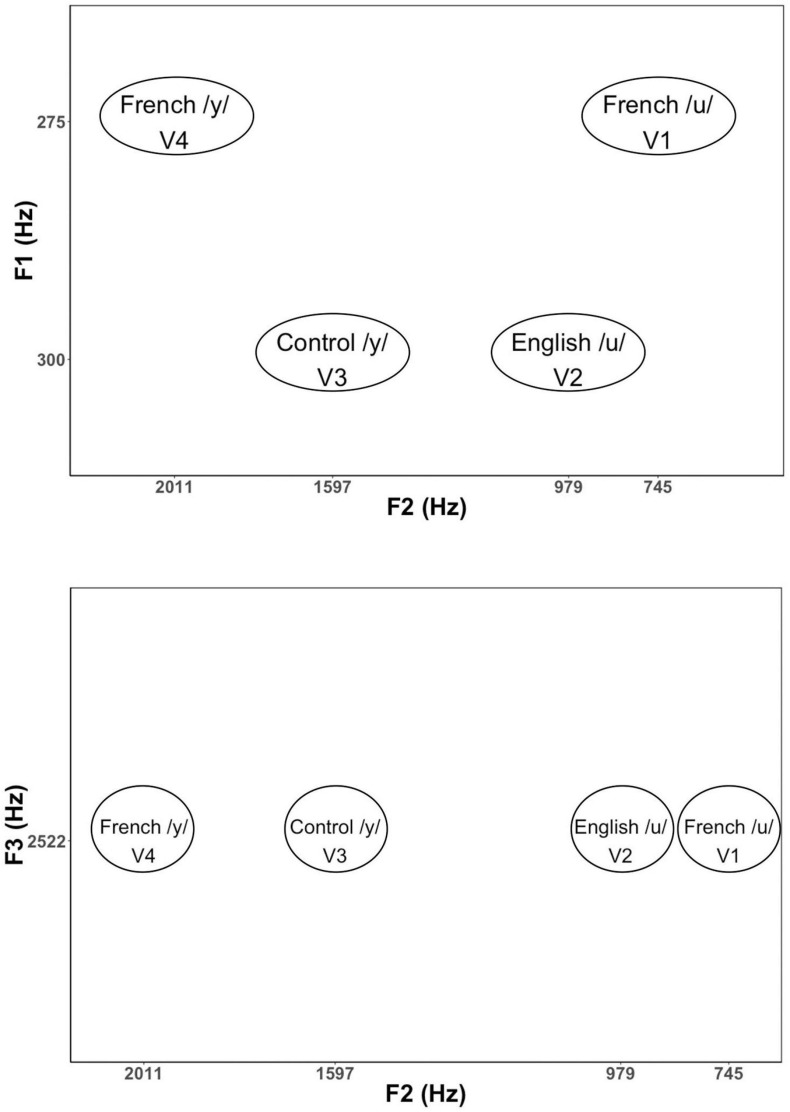
Formant values for the vowel stimuli in *F*_1_*–F*_2_ and *F*_2_*–F*_3_ spaces. *F*_1_ is related to the degree of constriction formed by the tongue in the vocal tract with lower *F*_1_ values corresponding to a tighter constriction formed by a higher tongue position. *F*_2_ is related to the location of the tongue constriction along the length of the vocal tract, with higher *F*_2_ values corresponding to constrictions closer to the lips. Lip rounding (lip compression and protrusion) increases vocal tract length, which in turn, has the effect of lowering all formants, especially *F*_3_.

**FIGURE 2:**
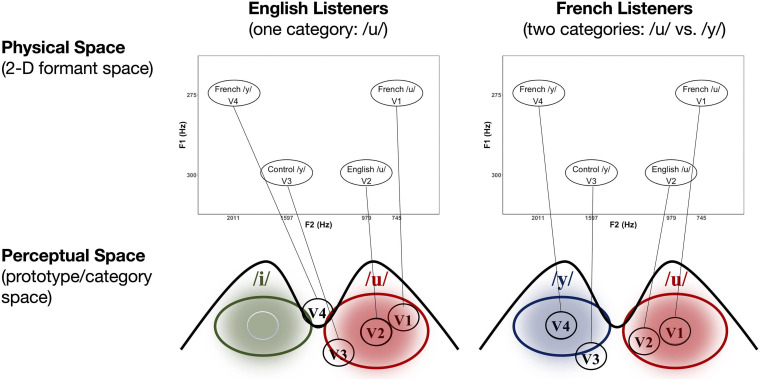
Schematic of the hypothesized relationship between physical (2D *F*_1_*–F*_2_ formant) space and perceptual (prototype/category) space in the case of the current vowel stimuli for both English (*left*) and French (*right*) listeners (see text for explanation).

Based on the results of these initial tests, we then selected 34 vowels to present to larger groups of English (*n* = 13) and French (*n* = 13) listeners in a subsequent experiment for identification and goodness ratings. This reduced stimulus set included 22 high back vowels targeting English /u/ and French /u/ vowel (*F*_1_ = 275 and 300 Hz; *F*_2_ = 4,548 to 979 Hz), and 12 high front vowels targeting English /i/ and French /i/ and /y/ (*F*_1_ = 275 and 300 Hz; *F*_2_ = 1,753 to 2,202 Hz). Note that we also synthesized two additional filler vowels (/o/[“oh”] and /∂/[“uh”]) to include in the stimulus set to provide some variation in vowel quality. This made it easier to assess whether participants were successful in identifying vowel quality differences using key words. The results of these tests were then used to select four vowel tokens (shown in Figure 1) for use in the current neurophysiological study: a good exemplar of French /u/ (*F*_1_ = 275 Hz, *F*_2_ = 745 Hz), a good exemplar of English /u/ (*F*_1_ = 300 Hz, *F*_2_ = 979 Hz), a good exemplar of French /y/ (*F*_1_ = 275 Hz, *F*_2_ = 2,011 Hz), and a poor exemplar of Canadian French /y/ (*F*_1_ = 300 Hz, *F*_2_ = 1,597 Hz). The selected variants of /u/ (V1 and V2) and /y/ (V3 and V4) were equally distant on the bark scale (Zwicker and Terhardt, 1980) along both the *F*_1_ and *F*_2_ dimensions. The French-prototypic/u/is the most focal between *F*_1_ and *F*_2_ (Figure 1, top panel), whereas the French-prototypic /y/ is the most focal between *F*_2_ and *F*_3_ (Figure 1, bottom panel). Table 1 gives the lower formants [*F*_1_–*F*_3_ (Hz)] and their corresponding bandwidths for each vowel stimulus. Figure 2 schematizes the underlying perceptual vowel spaces for each language group [English (top) vs. French (bottom)].

**TABLE 1:** Formant frequency and bandwidth (Hz) for the lower formants (F1, F2, and F3) for each vowel stimulus.

Stimulus	*F*_1_	*F*_2_	*F*_3_	*B*_1_	*B*_2_	*B*_3_
French/u/(V1)	275	745	2522	85	30	35
English/u/(V2)	300	979	2522	85	30	35
Control/y/(V3)	300	1597	2522	85	30	35
French/y/(V4)	275	2011	2522	85	30	35

### Procedure and Design

**FIGURE 3:**
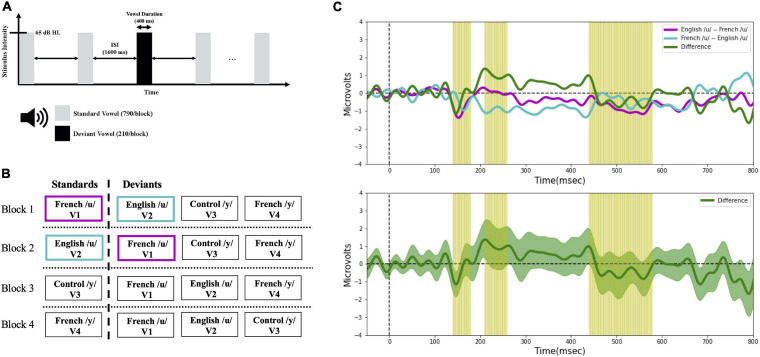
**(A)** Timeline of the stimulus presentation in the oddball task. **(B)** Schematic of the experimental design, and **(C)** example MMN waveforms. Four experimental blocks were presented to each participant in a randomized order. The color coding in panel **(B,C)** illustrates the ERP averaging technique applied in the current study. For example, to characterize the detection of V1 among V2 (i.e., V2 → V1 direction), the MMN (purple line) was calculated by subtracting the ERPs to French /u/ (V1) as deviant measured in a block where the English /u/ (V2) was the standard, from the ERPs to French /u/ (V1) as standard in a separate block. Conversely, to characterize the detection of V2 among V1 (i.e., V1 → V2 direction), the MMN (turquoise line) was calculated by subtracting the ERPs to English /u/ (V2) as deviant measured in a block where the French /u/ (V1) was the standard, from the ERPs to English /u/ (V2) as standard in a separate block. In this way, we obtained an average ERP for the standards and for the deviants that was unaffected by the physical characteristics of the stimuli, and only the oddball effect was present when comparing the standard and deviant waveforms. The same averaging technique was applied with the rest of the contrasts. To test for directional asymmetries in the MMN, difference waves (green line) were then computed by subtracting the MMN waveform for the two opposite stimulus orders (e.g., V2 → V1 vs. V1 → V2); this is shown in the bottom panel of 3C with the green shaded region representing the standard error.

Vowel perception was assessed with ERPs. The stimuli were presented across four different experimental blocks using a passive “multi-deviant oddball” task (Näätänen et al., 2004), as schematized in Figure 3. As shown in Figures 3A,B, different from the multi-feature paradigm, we also made sure there were at least two standards prior to each deviant. Further, rather than assigning the role of standard to one specific stimulus alone and the role of deviant to the other (remaining) stimuli of interest, as is typically done, all the vowels were presented both as standards and as deviants across the four different presentation blocks. This provided a way to control for potential differences in the N1 and P2 components (which overlap with the MMN) due to physical differences among the evoking stimuli. Within each block (in Figure 3B), a standard vowel alternated with three deviant vowels that differed in their first and second formant frequencies. The sequences of the four blocks were counter-balanced across subjects and language groups (English vs. French). The deviant and standard ratio was roughly 20:80 (each block contained 1,000 stimuli; 790 standards, 210 deviants [70 of each deviant vowel token]), and the inter-stimulus interval (ISI) was 1,600 ms. Within each block, deviants and standards were presented in a pseudo-random order ensuring that at least two standards preceded each deviant. During the recording sessions, participants sat in a comfortable armchair in an electrically shielded sound-attenuated booth and watched a silent movie under the instruction to ignore the stimuli. The stimulus output intensity was 65 decibels in hearing level (dB HL) and delivered to both ears through insert earphones (Etymotic Research). The experimental sessions lasted approximately 3.5 h including preparation time (approximately 40 min) and breaks (approximately 30 min).

### EEG Recording

EEG data were continuously recorded (500 Hz/32 bit sampling rate; Neuroscan Synamps2 amplifier) from 20 sites on the scalp with cap-mounted Ag-Ag/Cl electrodes (Electro-cap International, Inc., Eaton, OH, United States), based on the international 10–20 system of electrode placement: Fp1/2, F7/8, F3/4, T3/4, C3/4, T5/6, P3/4, O1/2, Fpz, Fz, Cz, Pz, and Oz. Eye movements and blinks were detected using electro-oculography (EOG). Vertical and horizontal EOG were recorded from bipolar electrodes placed above and below the left eye, and at the outer corner of each eye, respectively. All EEG electrodes were referenced against the right mastoid, and an electrode located between Fz and Fpz provided the ground. Electrode impedances were kept under 3 kOhm.

### Data Preprocessing and Analysis

EEG data were analyzed using Brain Vision Analyzer software (Brain Products GmbH, Germany), including offline band-pass filtering (0.5–30 Hz) and artifact rejection with a ±50 microvolts (μV) deviation criterion at all channels except for Fp1 and Fp2, which were clearly more affected by eye movements than the rest of the channels. Consequently, Fp1 and Fp2 were excluded from any further analysis and data processing. Artifact rejection resulted in data loss within a range of 3.45 and 11.09% across participants. Note that analyses with other band-pass settings (0.4–40, 0.4–100 Hz) were also computed. They resulted in the same findings reported here, but data included more noise.

**FIGURE 4:**
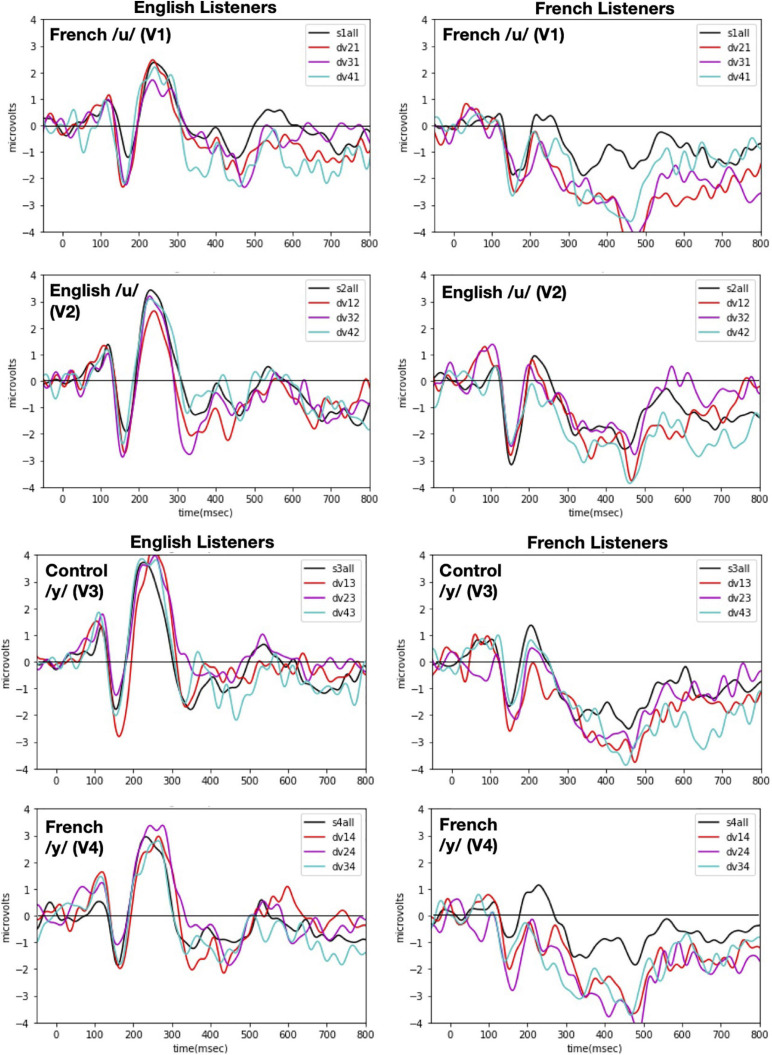
ERP responses recorded with each of the four vowel tokens as the standard (V1, V2, V3, and V4 from top panel to bottom panel) and for each language group (English Listeners: *left panels* and French Listeners: *right panels*). Each panel shows ERP responses recorded when one vowel (indicated in the left hand corner) was presented as the standard with each of the other vowels as the deviant. As indicated in the right hand corner, the deviants are plotted as different color lines and as a black line when all deviants are combined. Note: in labeling the waves, the first number designates the deviant vowel and the second number designates the standard, e.g., for dv21 and V2 is the deviant when V1 is the standard.

Event-related potentials were time-locked to vowel onset and were computed separately for the standard and deviant conditions of each vowel. Only the standard immediately preceding a deviant stimulus was included in the calculation of ERPs for standards in order to use the same number of stimuli in forming the standard and the deviant. The epochs were 850-ms long (-50 ms pre-stimulus and 800 ms post-stimulus onset) and were baseline corrected to the time period from 50 ms of pre-stimulus onset to 50 ms of post-stimulus activity. We opted for this baseline correction (instead of the typical –100 ms to 0) because there was a 50 ms (±4 ms) silence at the beginning of each sound file that we had realized once the experiment was completed. Future studies that wish to replicate our procedure should select time windows based on the actual stimulus onset, not the specific values reported here. Figure 4 shows the obtained ERP responses to each vowel token (V1 vs. V2 vs. V3 vs. V4) when presented in the contextual role of standard versus deviant for each language group [English (left panels) vs. French (right panels)].

#### Mismatch Negativity Response Analyses

A directional asymmetry is essentially a context effect, i.e., a difference found when the same stimuli are presented in a different order (or context). In behavioral discrimination tasks, directional asymmetries are assessed by comparing outcome measures (e.g., accuracy) across different orders (AB vs. BA). With ERP recordings, we can track neural processing of the individual stimuli within a sequence, which allows us to examine order/context effects at a deeper level. This was optimized by the current study design which ensured that subjects were presented each vowel within a pair as the deviant and also as a standard. For example, for vowel pair V1–V2, we can ask whether the processing of V1 is different (faster, stronger) when it follows V2 (serving as the deviant) than when it proceeds V2 (serving as the standard). With this in mind, MMN waveforms were calculated by subtracting standard ERPs from deviant ERPs of the same vowel, which allows us to target the context effects. For example, in the present study, to characterize the neural processing of the V1–V2 vowel pair we first calculated two MMN responses. The first MMN indexes the processing of V1 (in context of V2); to do so we took the ERPs recorded when V1 was the deviant (and V2 was the standard) minus the ERPs recorded when V1 was the standard (and V2 was the deviant); the result is plotted in Figure 3C (purple line). The second (or reverse) MMN indexes the processing of V2 (in the context of V1); to do so we took the ERPs recorded when V2 was the deviant (and V1 was the standard) minus the ERPs recorded when V2 was the standard (and V1 was the deviant); the result is plotted in Figure 3C (turquoise line).

We then conducted two types of analyses to examine whether asymmetries emerged in the neurophysiological responses to each of the vowel pairs: (i) a hypothesis-based analysis focused to examine possible directional effects on MMN responses in three *a priori* time windows identified in Molnar et al. (2013), and (ii) an exploratory temporal clustering analysis to reveal additional time windows where MMN responses may be asymmetrical within the entire epoch (Maris and Oostenveld, 2007). The time windows for the hypothesis-based analysis were selected by the visual inspection of the grand average waveforms (that included all the conditions across all the participants) on the Fz electrode, and corresponding to time points associated with the N1, MMN, and the late negativity. The visual inspection yielded three consecutive latency time windows: 140–180 ms, 210–260 ms, and 440–580 ms. For each vowel pair (e.g., V1–V2) we then computed the average MMN (V1 in context of V2) and reverse MMN (V2 in context of V1) value within each of these latency windows for each participant. These values were submitted to separate analyses of variance (ANOVAs)–latency window (140–180 vs. 210–260 vs. 440–580 ms) × direction (MMN vs. reverse MMN)–for each vowel pair (V1–V2, V1–V3, V1–V4, V2–V3, V2–V4, and V3–V4).

An additional hypothesis-based analysis, was conducted based on the amplitude of the MMN *difference wave* computed by subtracting the MMN waveforms for each vowel within a pair; the amplitude difference was computed at each latency window (140–180 vs. 210–260 vs. 440–580 ms). For example, as shown for vowel pair V1–V2 in Figure 3C, the green line represents the difference between the MMN for V1 (MMN above) and the MMN for V2 (reverse MMN above); the green shaded area in the lower panel represents the standard error, and the yellow shading corresponds to the three latency windows. Within each language group, MMN difference waves were calculated for each of the six vowel pairs (V1–V2, V1–V3, V1–V4, V2–V3, V2–V4, and V3–V4) and averaged across six electrode sites (F3, F4, Fz, C3, C4, and Cz). Within each latency window, we averaged the values across the time window for each participant and then submitted these values to separate mixed ANOVAs–latency window (140–180 vs. 210–260 vs. 440–580 ms) × language group (English vs. French)–for each vowel pair.

Finally, the exploratory temporal clustering analysis was conducted to determine whether there were any additional latency windows (not tested in the aforementioned analysis) in which the MMN difference waves were significantly different from 0 (the value expected if there is no order/context effect) within the entire epoch. This was a data-driven approach with no *a priori* hypotheses with regard to the latency window(s) where the difference waveforms would significantly differ from 0 μV (see Maris and Oostenveld, 2007). Specifically, we deployed the threshold-free cluster enhancement (TFCE) extension method (Smith and Nichols, 2009) which allows for improved sensitivity, but more interpretable output than traditional cluster-based thresholding. First, the TFCE values were generated by summing across a series of thresholds, thus avoiding selecting an arbituary threshold and then the p values for each time sample were calculated through permutation. The analysis was performed using the TFCE cluster test with a start = 0, step = 0.01, and 3,000 permutations, implemented in MNE python software (Gramfort et al., 2014).

#### Time-Frequency Analysis

Finally, to characterize theta activity (4–8 Hz) during vowel processing, we conducted time-frequency analyses on the ERPs of the standard trials for each vowel, using the multi-taper method implemented in MNE python (Gramfort et al., 2014). Similar to the MMN analyses, the ERPs of the standard trials were also averaged across the F3, F4, Fz, C3, C4, and Cz electrode sites. The mean theta-band activity for each vowel and each participant was further extracted by averaging across the time window between 0 and 600 ms and across the frequency band between 4 and 8 Hz. Repeated ANOVAs and paired-sample *t*-tests were then performed on the individual means to test for effects of formant proximity and stimulus prototypicality on theta activity. Greenhouse-Geisser corrections were applied when appropriate and partial eta-squared ($\eta_{p}^{2}$) was calculated for main effects and interactions.

## Results

### Mismatch Negativity Response Analyses

**FIGURE 5:**
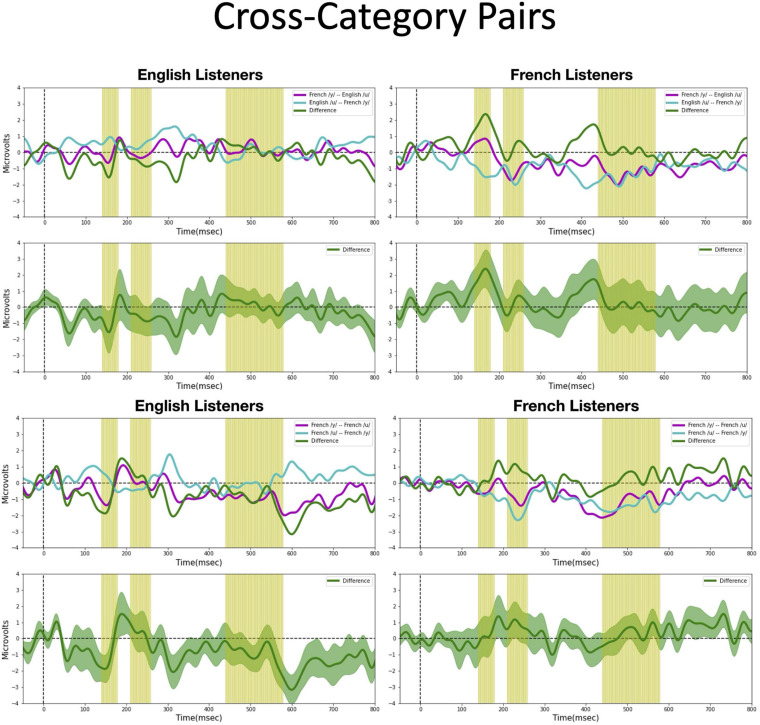
Mismatch negativities (as described in Figure 3C) for each stimulus type: *Cross-category* pairs, the mean MMNs for each stimulus presentation order (purple and turquoise lines) and difference waveform (green lines) (in the top half of each panel) and the difference waveform plotted with the standard error for the group (as the dark green shaded region) in the bottom half of each panel. The light green shading indicates the pre-selected time windows used in the hypothesis-based anayses. The MMNs are plotted for each language group wth English Listeners on the left and French Listeners on the right. The vowel pair for each MMN is indicated in the legend at right hand corner of each panel with the standard followed by the deviant (e.g., French /y/ – French /u/ denotes an MMN with French /u/ (V1) as the deviant in the context of French /y/ (V4) as standard).

**FIGURE 6:**
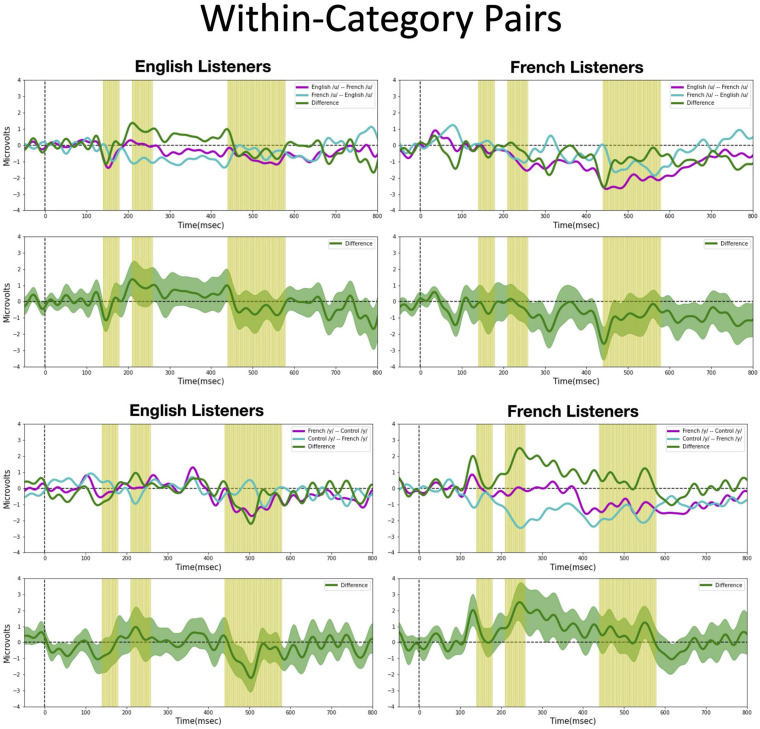
Mismatch negativities (as described in Figure 3C) for each stimulus type: *Within-category pairs*, the mean MMNs for each stimulus presentation order (purple and turquoise lines) and difference waveform (green lines) (in the top half of each panel) and the difference waveform plotted with the standard error for the group (as the dark green shaded region) in the bottom half of each panel. The light green shading indicates the pre-selected time windows used in the hypothesis-based anayses. The MMNs are plotted for each language group wth English Listeners on the left and French Listeners on the right. The vowel pair for each MMN is indicated in the legend at right hand corner of each panel with the standard followed by the deviant (e.g., French /y/ – French /u/ denotes an MMN with French /u/ (V1) as the deviant in the context of French /y/ (V4) as standard).

**FIGURE 7:**
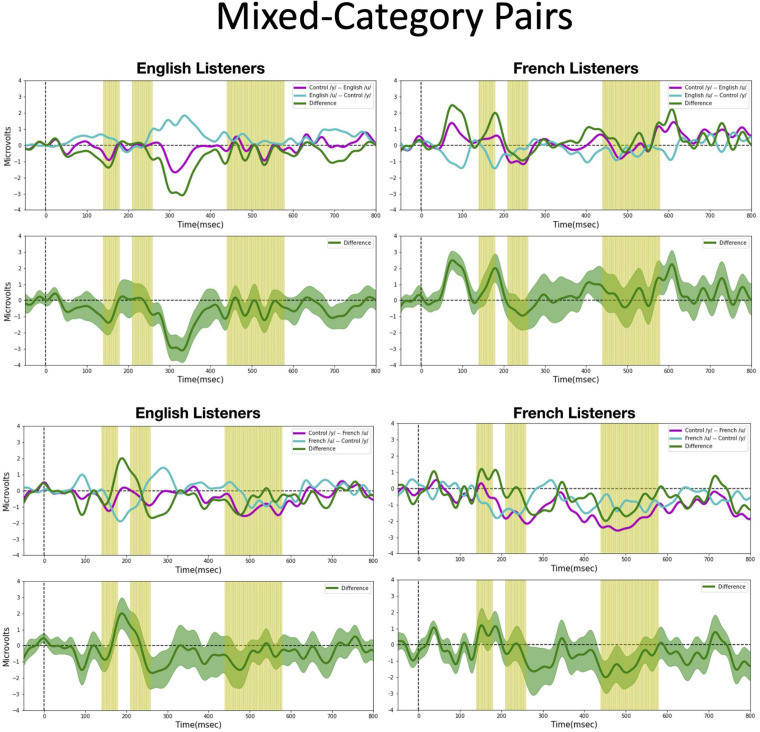
Mismatch negativities (as described in Figure 3C) for each stimulus type: *Mixed-category pairs*. the mean MMNs for each stimulus presentation order (purple and turquoise lines) and difference waveform (green lines) (in the top half of each panel) and the difference waveform plotted with the standard error for the group (as the dark green shaded region) in the bottom half of each panel. The light green shading indicates the pre-selected time windows used in the hypothesis-based anayses. The MMNs are plotted for each language group wth English Listeners on the left and French Listeners on the right. The vowel pair for each MMN is indicated in the legend at right hand corner of each panel with the standard followed by the deviant (e.g., French /y/ – French /u/ denotes an MMN with French /u/ (V1) as the deviant in the context of French /y/ (V4) as standard).

We assessed possible asymmetric patterns in the neurophysiological responses to all six vowel pairs within each language group and also cross-linguistically. Each of the six vowel pairs fell into one of three stimulus types: (1) *cross-category* pairs (V1–V4 and V2–V4) with relatively large acoustic differences; (2) *within-category* pairs (V1–V2 and V3–V4) with relatively small acoustic differences; and (3) *mixed-category* pairs (V1–V3 and V2–V3) with intermediate acoustic differences. Figures 5–7 show the MMN results averaged across six electrode sites (F3, F4, Fz, C3, C4, and Cz) and plotted as a function of language group (English vs. French) and vowel contrast (V1–V2; V2–V3;V3–V4; V1–V4; V2–V4; and V1–V3), grouped by stimulus type (*cross-category* vs. *within-category* vs. *mixed-category;* the dark green shaded area represents the standard error, and the yellow shading corresponds to the three latency windows). The ANOVA results comparing the MMN waves in each direction for each of the three *a priori* latency windows are summarized in Table 2; none of the vowel pairs showed a main effect of direction or an interaction effect (*p* > 0.05). The ANOVA results comparing the MMN *difference waves* for each language group and for each of the three *a priori* latency windows are summarized in Table 3; none of the vowel pairs showed any main or interaction effects (*p* > 0.05). The temporal cluster analyses also failed to reveal asymmetric MMN response in other temporal windows, except for a small region of the MMN response to the V2–V3 vowel pair (described below).

**TABLE 2:** Repeated measures an analysis of variance (ANOVA) on mismatch negativities (MMN) amplitude presented for each stimulus type (*cross-category* vs. *within-category* vs. *mixed-category*).

Effect	*F*	*df*	*p*	*np*^2^
***Cross-category pairs***				

*Vowel pair:* more-focal French/u/**(V1**) vs. more-focal French/y/(**V4**)				

Latency window	1.497	2	0.233	0.049
Direction	0.003	1	0.954	<0.001
Latency window × Direction	1.588	2	0.214	0.052

*Vowel pair:* less-focal English/u/(**V2**) vs. more-focal French/y/(**V4**)				

Latency window	3.206	2	0.058	0.100
Direction	0.101	1	0.753	0.003
Latency window × Direction	0.773	2	0.461	0.026
***Within-category pairs***				

*Vowel pair:* more-focal French/u/(**V1**) vs. less-focal English/u/(**V2**)				

Latency window	3.297	2	0.054	0.102
Direction	0.191	1	0.665	0.007
Latency window × Direction	1.308	2	0.277	0.043

*Vowel pair:* less-focal/control/y/(**V3**) vs. more-focal/control/y/(**V4**)				

Latency window	6.092	2	0.007**	0.174
Direction	0.491	1	0.489	0.017
Latency window × Direction	2.326	2	0.112	0.074
***Mixed-category pairs***				

*Vowel pair:* less-focal English/u/(**V2**) vs. more-focal control French/y/(**V3**)				

Latency window	0.409	2	0.614	0.014
Direction	0.1	1	0.754	0.003
Latency window × Direction	0.414	2	0.635	0.014

*Vowel pair:* more-focal French/u/(**V1**) vs. more-focal control French/y/(**V3**)				

Latency window	1.239	2	0.296	0.041
Direction	0.345	1	0.561	0.012
Latency window × Direction	2.212	2	0.119	0.071

Mismatch negativities for each vowel pair were analyzed using an ANOVA with the main factors of latency window (140–180 vs. 210–260 vs. 440–580 ms), direction (MMN vs. reverse MMN), and the interaction effect. Shown are the *F* value, the degrees of freedom, *p* value, and partial-eta-squared value for each effect, *p < 0.05; **p < 0.01.

**TABLE 3:** Mixed ANOVA on MMN Amplitude difference (MMN minus reversed MMN) for each stimulus type (*cross-category* vs. *within-category* vs. *mixed-category*).

Effect	*F*	*df*	*p*	*np*^2^
***Cross-category pairs***				

*Vowel pair:* more-focal French/u/**(V1**) vs. more-focal French/y/(**V4**)				

Latency window	1.542	2	0.224	0.052
Language group	0.718	1	0.404	0.025
Latency window × Group	0.148	2	0.856	0.005

*Vowel pair:* less-focal English/u/(**V2**) vs. more-focal French/y/(**V4**)				

Latency window	0.802	2	0.451	0.028
Language group	1.308	1	0.262	0.045
Latency window × Group	2.067	2	0.137	0.069
***With in-category pairs***				

*Vowel pair:* more-focal French/u/(**V1**) vs. less-focal English/u/(**V2**)				

Latency window	1.279	2	0.285	0.044
Language group	0.354	1	0.557	0.012
Latency window × Group	0.349	2	0.683	0.012

*Vowel pair:* less-focal/control/y/(**V3**) vs. more-focal/control/y/(**V4**)				

Latency window	2.253	2	0.12	0.074
Language group	1.554	1	0.223	0.053
Latency window × Group	0.094	2	0.894	0.003
***Mixed-category pairs***				

*Vowel pair:* less-focal English/u/(**V2**) vs. more-focal control French/y/(**V3**)				

Latency window	0.431	2	0.618	0.015
Language group	0.823	1	0.372	0.029
Latency window × Group	2.176	2	0.132	0.072

*Vowel pair:* more-focal French/u/(**V1**) vs. more-focal control French/y/(**V3**)				

Latency window	2.185	2	0.122	0.072
Language group	0.002	1	0.965	<0.001
Latency window × Group	0.652	2	0.523	0.023

*Mismatch negativity difference scores for each vowel pair were analyzed using ANOVA with the main factors of latency window (140–180 vs. 210–260 vs. 440–580 ms), language group (English vs. French), and the interaction effect. Shown are the *F* value, the degrees of freedom, *p* value, and partial-eta-squared value for each effect.*

*Cross-category pairs* (Figure 5). For the *cross-category* (/u-y/) vowel pairs (V1–V4 and V2–V4), recall the following NRV predictions: (1) for non-native (English) listeners, the MMN response will be greater (and/or earlier) when the more focal variants (V1 and V4) serve as the deviant stimulus, whereas (2) for native (French) listeners, vowel processing for *cross-category* pairs is predicted to be more symmetrical. No reliable asymmetry was found for either vowel pair in any of the pre-selected latency windows for either language group. No additional significant time windows were revealed by the exploratory temporal cluster analyses.

*Within-category pairs* (Figure 6). For the *within-category* pairs (V1–V2 and V3–V4), recall that NRV predicts that the MMN response will be greater (and/or earlier) when the more focal variants (V1 and V4) serve as the deviant stimulus compared to the reverse, regardless of native language. In contrast, NLM predicts that the MMN response will be greater (and/or earlier) when the more prototypical variant of a native vowel category serves as the deviant stimulus compared to the reverse. According to this view, the MMN is expected to be stronger in the English listeners when V2 serves as the deviant among V1 standards, whereas for the French listeners, the MMN is expected to be stronger in the French listeners when V1 serves as the deviant among V2 standards and when V4 serves as the deviant among V3 standards. Although there was a trend for the MMN responses to pattern in the manner predicted by NLM across both language groups for the V1–V2 vowel pair, these differences did not reach statistical significance in any of the predetermined latency windows. However, for the V3–V4 pair in the 210–260 ms time window, the difference wave was significantly above 0 [*t*(14) = 2.20, *p* = 0.04], such that the MMN was stronger in the French group when V4 was the deviant compared to when V3 was the deviant). This finding is consistent with both NRV and NLM since the French prototypic /y/ (V4) is more focal (between *F*_2_ and *F*_3_) than the non-prototypic French /y/ (V3). No additional significant time windows were revealed by the cluster analyses.

*Mixed-category pairs* (Figure 7). For the *mixed-category* pairs (V2–V3 and V1–V3), NRV predicts that the MMN response will be greater when the more focal stimulus (V1 and V2) serves as the deviant compared to the reverse. NLM would predict that the MMN should be greater in the English group (in which V1, V2, and V3 are perceived as variants of the native /u/ category) when V2 serves as the deviant among V3 standards since V2 is the category prototype. Here, no significant asymmetries were found for either vowel contrast for either language group in the analyses using pre-selected time windows. However, the exploratory, temporal cluster permutation tests revealed an asymmetric MMN response between V2 and V3 or both English and French listeners but in opposite directions. For the English group, the time windows between 292–300 and 324–334 ms in the difference wave were significantly below 0 (*p* < *0.05*), whereas for the French group, the time window in the difference wave between 66 and 104 ms was significantly above 0 (*p* < *0.05*). These asymmetries do not align across language groups; for the English group, the MMN was stronger when the stimuli were presented in the direction going from V3 to V2, whereas for the French group, the MMN was stronger when the stimuli were presented in the direction going from V2 to V3. While the English results may be interpreted as a prototype effect, neither NRV nor NLM explicitly predicted the directional effect observed in the French group.

Overall, both the hypothesis-based and the exploratory analyses failed to provide evidence that the MMN responses to these vowel stimuli are asymmetric. The threshold-free cluster enhancement method that we applied is designed to isolate meaningful, non-random effects, through a data-driven approach. Given that the time windows tagged to have significant asymmetries using this method are quite short and also do not temporally align across the language groups these results should be interpreted with caution. These MMN findings alone do not provide sufficient evidence to draw conclusions about the neural processes that underlie the asymmetries in vowel processing that are observed in behavior.

### Theta Rhythms

**FIGURE 8:**
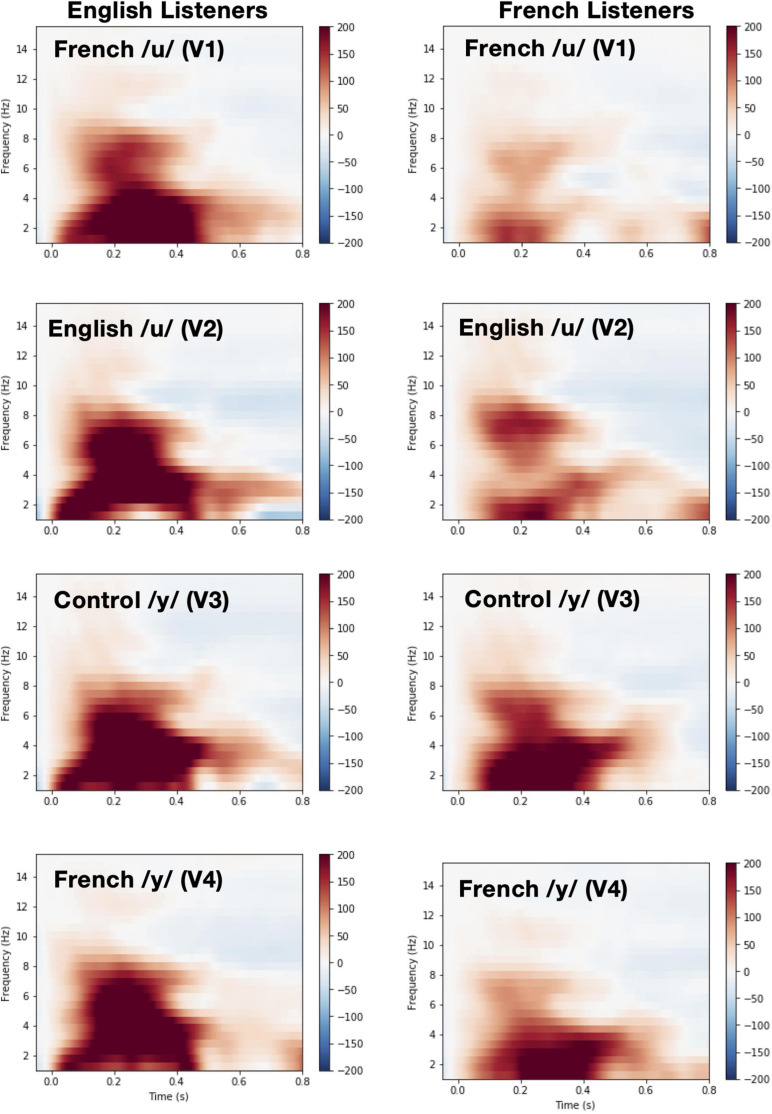
Time-Frequency plots showing brain oscillations for each vowel during pre-deviant standard trials (averaged across the F3, F4, Fz, C3, C4, and Cz electrode sites, 0–600 ms time window and between 4 and 8 Hz) and for each language group [English (*left panel*) vs. French (*right panel*)].

**FIGURE 9:**
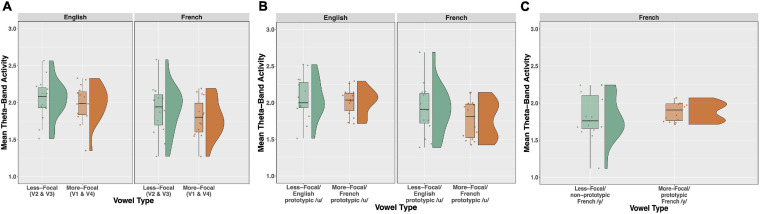
Raincloud plots, which combine boxplots and split-half violin plots, of theta-band activity elicited in response to the vowel stimuli. **(A)** Theta activity for the relatively less-focal vowels (V2 and V3) versus more-focal vowels (V1 and V4) plotted as a function of each language group (English vs. French). **(B)** Theta for the more-focal /u/ (V1) versus less-focal /u/ (V2) for each language group. **(C)** Theta for the less-focal, non-prototypic /y/ (V3) vs. more-focal prototypic /y/ (V4) for French listeners. There was a main effect of vowel type in panels **(A,B)**, such that theta was lower for the more-focal vowel variants, and this effect was significant within each language group (see text for further explanation).

Time-frequency plots showing brain oscillations (averaged across the F3, F4, Fz, C3, C4, and Cz electrode sites, 0–600 ms time window) for each vowel stimulus (V1 vs. V2 vs. V3 vs. V4) and language group (English vs. French) are given in Figure 8. We tested two specific hypotheses that derive from NRV and NLM, respectively: (1) mean theta activity, which has been argued to reflect attention and processing efficiency during speech processing (Bosseler et al., 2013), will be lower for the relatively more-focal vowel stimuli (V1 and V4) compared to the relatively less-focal vowel stimuli (V2 and V3); and (2) mean theta activity will be lower for the more-prototypical native-language vowel stimuli compared to the less-prototypical vowel stimuli. We first examined whether mean theta-band activity was influenced by formant proximity, independent of variation in stimulus prototypicality, in a mixed ANOVA with language group (English vs. French) as a between-subjects factor and vowel type [more-focal (V1–V4) vs. less-focal (V2–V3)] as a within-subjects factor. Figure 9A shows the theta rhythm results for the less-focal versus the more-focal vowels for each language group. Consistent with the predictions derived from NRV, there was a main effect of vowel type [*F*(1,28) = 5.786, *p* = 0.023, *$\eta_{p}^{2}$* = 0.171], such that theta activity was lower for the more-focal vowels compared to the less-focal vowels. Neither the effect of language group [*F*(1,28) = 2.855, *p* = 0.102, *$\eta_{p}^{2}$* = 0.093] nor the interaction effect [*F*(1,28) = 0.676, *p* = 0.418, *$\eta_{p}^{2}$* = 0.024] reached statistical significance.

Next, to test whether mean theta-band activity was influenced by stimulus prototypically, we conducted a mixed ANOVA with language group (English vs. French) as a between-subjects factor and vowel type [more-focal (V1) vs. less-focal (V2)] as a within-subjects factor. Figure 9B shows the theta rhythm results for the more-focal /u/ (V1) versus the less-focal /u/ (V2) for each language group. These results of the analysis revealed that there was a main effect of vowel type [*F*(1,28) = 6.871, *p* = 0.014, *$\eta_{p}^{2}$* = 0.197], such that theta activity was lower for the more-focal variant (*M* = 1.85; *SD* = 0.29) compared to the less-focal variant (*M* = 1.98; *SD* = 0.32). Neither the effect of language group [*F*(1,28) = 2.684, *p* = 0.113, *$\eta_{p}^{2}$* = 0.087] nor the interaction effect [*F*(1,28) = 0.040, *p* = 0.844, *$\eta_{p}^{2}$* = 0.001] reached statistical significance. This finding aligns with the prior analysis showing that theta activity is lower during the processing of more-focal vowel stimuli, regardless of variation in category “goodness.” It is important to note that because the magnitude of this difference was not greater for the French group compared to the English group, it further demonstrates that the aligned effects of focalization and stimulus prototypicality are not greater than effects of focalization alone. In a final analysis, we compared theta activity for the less-focal, non-prototypic /y/ (V3) vs more-focal prototypic /y/ (V4) for French listeners only because this is not a within-vowel category difference for English listeners. These results are shown in Figure 9C. Here, theta activity did not significantly differ between V3 and V4 [*t*(14) = 1.052, *p* = 0.310], indicating that the aligned effects of focalization and prototypicality is weak for this vowel pair.

## Discussion

In the current research we asked the following question: can we observe directional asymmetries in the neurophysiological correlates of vowel processing, and, if so, can these effects be attributable to processing differences related to generic phonetic biases, as predicted by the NRV framework (Polka and Bohn, 2011), and/or stimulus prototypicality, as predicted by the NLM model (Kuhl, 1991; Kuhl et al., 2008)? To address this, we focused on the MMN and theta brain rhythms elicited in response to cross-category and within-category vowel pairs by English- and French-speaking adults. Recent behavioral research using very similar /u/ stimuli (Masapollo et al., 2015, 2017a; Liu et al., 2021) has shown directional asymmetries that are consistent with NRV when acoustic differences are relatively large, and that follow NLM predictions when acoustic differences are relatively small and very close to the location of a native category prototype in psychophysical space. The present study extends this work by showing that the pre-attentive MMN response may not be a reliable neurophysiological correlate of the asymmetric behavioral vowel discrimination. Rather, the data suggest that asymmetries, as revealed by theta oscillatory activity, may reflect differences in neural processing efficiency for vowels with varying degrees of formant convergence.

One possible explanation for the lack of robust directional differences in the MMN responses (shown in Figures 5–7 and Tables 2, 3) is that the present neurophysiological testing procedures require less cognitive demands than the prior behavioral paradigms (Masapollo et al., 2015, 2017a; Liu et al., 2021). More specifically, the ERPs were elicited by *passive* listening to the vowel stimuli; participants were not required to actively attend to the stimuli or make overt behavioral responses. In fact, they were distracted by a silent movie and instructed to ignore the auditory stimuli. Thus, the failure to observe asymmetric MMN responses could be due to the minimal processing demands in the ERP task in comparison to behavioral discrimination tasks where perceptual asymmetries are observed. For example, the AX discrimination task used by Masapollo et al. (2017a) required participants to attend to subtle acoustic differences in the vowel stimuli, to encode and buffer this information across a 1,500 ms delay, and then arrive at a “same” or “different” judgment within a brief time interval.

Although the MMN response failed to reveal asymmetries, the neural efficiency data suggests that cognitive demands are decreased when listeners process relatively more focal compared to relatively less focal vowels. Across both language groups, theta band activity was lower in response to the more focal variants but not to the more prototypic variants (Figure 9). This is consistent with theoretical accounts that vowels with a greater degree of formant convergence are easier to process and are more stable in phonological working memory compared to less focal vowels (Schwartz and Escudier, 1989; Schwartz et al., 1997, 2005; Polka and Bohn, 2011; Masapollo et al., 2017a).

The results also revealed a trend (albeit non-significant) for theta-band activity to be lower for the French listeners than the English listeners (Figure 9). These cross-linguistic processing differences align with behavioral data showing that French listeners are more sensitive to acoustic variations in this part of vowel space (Masapollo et al., 2017a,b). This may be attributable to differences in the vowel systems of English and French. More specifically, French has a richer inventory of high vowels (/i y u/) than English (/i u/; see, Escudero and Polka, 2003). Thus, this high region of the vowel space is denser in French than in English, which may explain French listeners’ enhanced sensitivity to spectral differences in this part of the articulatory/acoustic vowel space. An alternative, but not mutually exclusive, explanation is that among our French subjects, who are functionally monolingual, at least some had experienced passive exposure to spoken English, and also heard English-accented French, while growing up and living in Quebec. In comparison, our English participants were university students from outside of Quebec studying in Montreal, they are less likely to have gained passive experience listening to French. Nevertheless, the current theta-band activity results raise the possibility that the roots of these cross-linguistic differences in behavioral discrimination lie in more efficient use of underlying neural mechanisms.

The present neurophysiological study builds on prior behavioral studies that seek to understand universal and experience-dependent factors that interact to shape vowel perception across the lifespan. Our findings add to an existing body of research that has employed the MMN or other neural measures to assess asymmetric patterns in speech processing. Unlike the present study, much of the prior MMN work related to asymmetries was motivated and designed to assess theoretical perspectives on phonological processing, typically focusing on evaluating models that posit different feature-based approaches such as abstract/under-specification versus detailed/full specification views (Eulitz and Lahiri, 2004; Cornell et al., 2011, 2013; Scharinger et al., 2012; Hestvik and Durvasula., 2016; Hojlund et al., 2019). For example, Eulitz and Lahiri (2004) examined German adults’ discrimination of several *native* vowel contrasts (i.e., /e/-/o/, /ø/-/o/, /e/-/ø/) in a passive oddball task, using event-related potentials. Not-surprisingly, listeners showed mismatch negativity (MMN) responses to all of the contrasts. However, the results also indicated that for the /ø/-/o/ contrast, listeners showed larger and earlier MMN amplitudes when /ø/ was the deviant, compared to when /o/ was the deviant. Listeners did not show asymmetric MMN responses for either of the other two vowel contrasts. The authors interpret these findings as suggesting that /ø/-/o/ has a different *phonological* status than the other vowel contrasts tested, and that this difference in phonological representation might explain the neural processing differences. Specifically, they postulate that the place of articulation feature [coronal] is universally absent or “underspecified” from phonemic representations in the lexicon–for vowels and consonants alike. In this view, the listeners tested in their oddball task may have elicited a larger and earlier MMN response when /ø/ was presented as a deviant in a train of /o/ standards, compared to the reverse, because /ø/ has an underspecified [coronal] place of articulation. These results raise the possibility that the structure of phonological representations for vowels in the lexicon must be considered along with formant proximity (Polka and Bohn, 2011) to account for what is currently known about native-language vowel processing in adults.

In general, though, prior research on asymmetries in speech processing has focused more on consonants rather than vowels and has not always included behavioral findings, making it unclear how to interpret some MMN findings. More importantly, prior MMN studies investigating asymmetric patterns in vowel processing have typically not considered specific perceptual or phonetic biases. Accordingly, vowel contrasts/stimuli used often do not allow for clear predictions à la NRV or NLM and the acoustic phonetic details (e.g., *F*_3_ values) needed to consider NRV predictions are often not reported. Some methodological choices also make it difficult or impossible to connect reported MMN findings with predictions based on more perceptually oriented models like NRV or NLM. For example, most prior MMN studies present stimuli using ISI (inter-stimulus interval) values that are very short compared to the values used in perception studies; this is problematic given that an NRV bias is not expected when the ISI is short because this promotes low-level acoustic biases rather than phonetic encoding of vowels (Masapollo et al., 2018b).

Similarly, the present study was also not motivated or designed to assess conceptual views on phonological processing. We choose (sub-lexical) stimuli and task conditions that allow us to make clear predictions about the role of focalization and prototypicality in neural speech processing, but these choices do not permit clear predictions about competing feature-based phonological models, such as the FUL (Featurally Underspecified Lexicon) model (Eulitz and Lahiri, 2004). Importantly, although our work has a somewhat narrower focus, we do not assume that the phonetic biases hypothesized within NRV or NLM are incompatible with phonological processing models, including more abstract views like FUL that propose under-specification of phonological representations. Rather, we expect that phonetic biases must eventually align with and support mature phonological processing. Explaining how this developmentally unfolds is a critical goal of future research. Achieving this goal will require us to design studies that assess divergent theoretical perspectives in an integrated rather than a parallel fashion. Going forward, it will also be important to examine and control relevant low-level acoustic parameters and task parameters. This is needed to clarify when we are measuring simple order or context effects that are possible procedural artifacts and when we are measuring aspects of stimulus salience that also operate under natural speech processing conditions.

We clearly need further research into the relations between behavioral and neural levels of vowel processing in cross-linguistic studies with adults as well as infants early in development. Several recent ERP studies (Slabu et al., 2012; Zhao et al., 2019) focusing on *subcortical* auditory processing of speech sounds in adults have found evidence to corroborate some aspects of the existing behavioral data on directional asymmetries (Masapollo et al., 2017a). In the Zhao et al. study, English listeners were presented with resynthesized (shortened) versions of the less-focal/English-prototypic /u/ and more-focal/French-prototypic /u/ identified by Masapollo et al. (2017a) in a passive listening task. The researchers recorded the frequency-following response (FFR) with the two stimuli arranged in oddball and reversed-oddball blocks. It is generally assumed that the FFR reflects the encoding of acoustic energy in the fundamental frequency (*F*_0_) and lower harmonics of vowel stimuli (Krishnan, 2002; Skoe and Kraus, 2010; Bidelman et al., 2013; Bidelman, 2018). Accordingly, Zhao and colleagues used the FFR as a way to assess whether there was more robust neural encoding in the frequency range of the *F*_1_ region for the more focal versus the more prototypical variant of the native vowel category/u/. They found that English listeners show enhanced power at the frequencies corresponding to *F*_1_ when listening to the more-focal/French prototypic /u/, but only when it served as the deviant stimulus. However, this pattern was not found in the neural encoding of *F*_1_ in response to the less-focal/English prototypic/u/; for the less focal /u/ the neural encoding was comparable regardless of whether the vowel served as the standard or deviant stimulus. These findings suggest that focality impacts the neural encoding of vowels.

While Zhao et al.’s (2019) results revealed an intriguing parallel between subcortical ERP and behavioral measures of auditory vowel processing, the precise nature of the relations between these measures remain unclear in part because they were obtained under very different task demands and stimulus presentation speeds. The behavioral perceptual tasks utilized by Masapollo et al. (2017a) and Liu et al. (2021) were more demanding in terms of attention and memory than the passive oddball task used by Zhao et al. (2019). In the AX discrimination tasks, listeners were required to make overt similarity judgements about pairs of stimuli separated by relatively long inter-stimulus intervals (ISIs) (i.e., 1,500 ms), whereas in the ERP task, listeners attended to a silent video while passively listening to trains of vowel stimuli separated by very short ISIs (i.e., ∼50 ms).

In future work, it will be informative to explore how task and stimulus factors modulate neurophysiological responses to vowel stimuli. If asymmetries evident in behavior are supported by pre-attentive processes, this may be more clearly observed in the standard passive task using a more typical short ISI. If, however, asymmetries are boosted by active and attentive processing of phonetic structure in the speech signal, as posited by NRV, then asymmetric patterns in brain activity should be more prominent during active processing tasks. Given that vowel perception asymmetries have been observed during unimodal (visual-only) as well as bimodal (audio-visual) vowel processing (Masapollo et al., 2017b, 2018a; Masapollo and Guenther, 2019), it will also be informative to record and compare neurophysiological responses in adults using visual-only and auditory-visual vowel stimuli. This could bring new insights into the brain networks linked to the processing of speech signals as phonetic units.

Apart from those differences in task demands, it is also unclear whether the subcortical ERP measures themselves directly relate to asymmetries in vowel perception documented at the behavioral level. Although the functionality and origins of the FFR are a topic of active debate, there is mounting evidence that the FFR arises from activity generated by both cortical and subcortical structures along the auditory pathway, and reflects pre-attentive neural tracking of sustained periodic information; namely, the fundamental frequency and higher harmonics in vowels (Skoe et al., 2013; Coffey et al., 2016; Tichko and Skoe, 2017; Bidelman, 2018). However, even if we assume this to be the case, we are still left with the question of whether and how such components from deep in the brainstem relate to cortical levels of processing and perception (for recent discussion, Zhao and Kuhl, 2018).

Finally, although the preponderance of evidence suggests that asymmetries in vowel perception derive from cognitive encoding strategies involving attention and working memory rather than general auditory processes (Polka and Bohn, 2011; Masapollo et al., 2017b, 2018b), analogous effects have also been reported with non-speech tonal analogues of vowels that approximate some of the temporal characteristics of naturally-produced /u/ vowels executed with more versus less extreme lip gestures (Masapollo et al., 2019). While such findings may be interpreted as evidence that asymmetries reflect (at least in part) general auditory processing biases, it is also possible that they reflect fundamentally different types of processes than those captured using speech stimuli (see also, Bishop et al., 2005; Timm et al., 2011). Future studies could directly compare subcortical and cortical responses to vowels and non-speech tones that track the center of the formant paths of the vowels. Such analyses will help to further uncover how adult and infant brains process vowels.

## Data Availability Statement

The data and analysis code supporting the conclusions of this article is available at: https://osf.io/5ubsh/?view_only=08560bc5fdac4d7f82af7418a6820d7d.

## Ethics Statement

The studies involving human participants were reviewed and approved by McGill University, Faculty of Medicine Institutional review board. The patients/participants provided their written informed consent to participate in this study.

## Author Contributions

MMo, TCZ, and MMa analyzed the data. LP, TCZ, and MMa wrote the manuscript. LP and MMo designed the ERP experiment. MMo conducted the ERP experiment. All authors contributed to the article and approved the submitted version.

## Conflict of Interest

The authors declare that the research was conducted in the absence of any commercial or financial relationships that could be construed as a potential conflict of interest.
